# Cellular basis for singing motor pattern generation in the field cricket (*Gryllus bimaculatus* DeGeer)

**DOI:** 10.1002/brb3.89

**Published:** 2012-09-04

**Authors:** Stefan Schöneich, Berthold Hedwig

**Affiliations:** Department of Zoology, University of CambridgeDowning Street, Cambridge, CB2 3EJ, U.K

**Keywords:** Acoustic communication, central pattern generator, identified interneuron, insect, species-specific motor pattern, stridulation

## Abstract

The singing behavior of male crickets allows analyzing a central pattern generator (CPG) that was shaped by sexual selection for reliable production of species-specific communication signals. After localizing the essential ganglia for singing in *Gryllus bimaculatus*, we now studied the calling song CPG at the cellular level. Fictive singing was initiated by pharmacological brain stimulation. The motor pattern underlying syllables and chirps was recorded as alternating spike bursts of wing-opener and wing-closer motoneurons in a truncated wing nerve; it precisely reflected the natural calling song. During fictive singing, we intracellularly recorded and stained interneurons in thoracic and abdominal ganglia and tested their impact on the song pattern by intracellular current injections. We identified three interneurons of the metathoracic and first unfused abdominal ganglion that rhythmically de- and hyperpolarized in phase with the syllable pattern and spiked strictly before the wing-opener motoneurons. Depolarizing current injection in two of these opener interneurons caused additional rhythmic singing activity, which reliably reset the ongoing chirp rhythm. The closely intermeshing arborizations of the singing interneurons revealed the dorsal midline neuropiles of the metathoracic and three most anterior abdominal neuromeres as the anatomical location of singing pattern generation. In the same neuropiles, we also recorded several closer interneurons that rhythmically hyper- and depolarized in the syllable rhythm and spiked strictly before the wing-closer motoneurons. Some of them received pronounced inhibition at the beginning of each chirp. Hyperpolarizing current injection in the dendrite revealed postinhibitory rebound depolarization as one functional mechanism of central pattern generation in singing crickets.

## Introduction

In all animals, including man, rhythmically repetitive movements such as breathing, walking, or flying are driven by central pattern generator (CPG) networks of the central nervous system (CNS) (Delcomyn 1980). Systematic identification of CPG neurons and their synaptic connections revealed the functional circuitry of several small CPG networks (Marder et al. 2005). By analyzing cellular and network mechanisms underlying rhythmic pattern generation (Marder and Calabrese 1996; Selverston 2010), invertebrate model systems provided the conceptual foundation to understand how similar circuits may operate in more complex brains (Pearson 1993; Yuste et al. 2005; Clarac and Pearlstein 2007; Harris-Warrick 2010).

The rhythmic output of a CPG originates either from emergent network properties deriving from mutual synaptic coupling between interneurons (e.g., locomotory rhythms, Satterlie 1985), endogenous bursting properties of individual pacemaker cells (e.g., respiration in mammals, Paton et al. 2006), or as a combination of both mechanisms (e.g., leech heart, Cymbalyuk et al. 2002). Even though all CPG circuits can endogenously produce rhythmic motor patterns without sensory feedback or other rhythmic inputs, most CPGs are nevertheless extensively modulated by sensory feedback (Pearson 1995; Beenhakker et al. 2005; Büschges and Gruhn 2008) and neuromodulators (Dickinson 2006; Harris-Warrick 2011) to allow flexible adjustment to varying external conditions (Pearson 2000).

Besides locomotion, intraspecific communication with stereotypically repeated visual, vibratory, or acoustic signals is also based on centrally generated rhythmic motor activity. Crickets are a well-established model system for studying principles of species-specific acoustic communication (e.g., Huber 1962; Hoy and Paul 1973; Schildberger 1994; Poulet and Hedwig 2006; Schöneich and Hedwig 2010; Grace and Shaw 2011). The males produce a genetically fixed calling song pattern (Bentley and Hoy 1972) that has to match the sharply tuned auditory recognition mechanism of the conspecific females (Pollack and Hoy 1979; Weber and Thorson 1989). However, the neuronal network that generates the singing motor pattern is still a virtually uncharted area (reviews: Kutsch and Huber 1989; Elsner 1994; Gerhardt and Huber 2002). From his pioneering studies, Huber (1955, 1960) initially concluded that the singing pattern is generated in distinct neuropiles of the cricket brain. Following experiments, however, demonstrated that the head ganglia are not directly involved in the central pattern generation of the crickets calling song (Otto 1971; Kutsch and Otto 1972). A pair of descending brain neurons merely controls the singing behavior by serving as command neurons that activate the singing CPG with their tonic spike discharge (Hedwig 2000). As the mesothoracic ganglion houses the motoneurons, which are driving the sonorous wing movements, for some decades it was surmised that the singing CPG is also located in this ganglion (review: Kutsch and Huber 1989). Recent studies, however, demonstrated that the neural network that generates the singing motor pattern spans from the metathoracic (Hennig and Otto 1995) to the first unfused abdominal ganglion (Schöneich and Hedwig 2011), and preliminary experiments reported an ascending singing interneuron in the first unfused abdominal ganglion, which elicited and reset the singing motor pattern when stimulated with intracellular current injection (Schöneich and Hedwig 2011).

To further investigate the neuronal mechanisms underlying singing pattern generation in the cricket CNS, here we quantitatively analyzed the rhythmic spike activity of wing-opener and wing-closer motoneurons during fictive singing, and we systematically probed the ventral nerve cord with sharp microelectrodes to identify and analyze interneurons of the singing network.

## Materials and Methods

### Animals

Male Mediterranean field crickets (*Gryllus bimaculatus* DeGeer) were selected 5–20 days after their final molt from the colony at the Department of Zoology (University of Cambridge, U.K.) and maintained under crowded conditions at 28°C on a 12h:12h light:dark cycle. Nearly 400 crickets were used for this study. After the preparation, about 50% sang for extended periods of time to allow exploring the ventral nerve cord with intracellular recordings and to narrow down the regions of the singing network. Presented data are based on recordings in 38 crickets. Experiments were carried out at 20–25°C and complied with the principles of Laboratory Animal Care.

### Preparation and pharmacological brain stimulation

**Figure 1:**
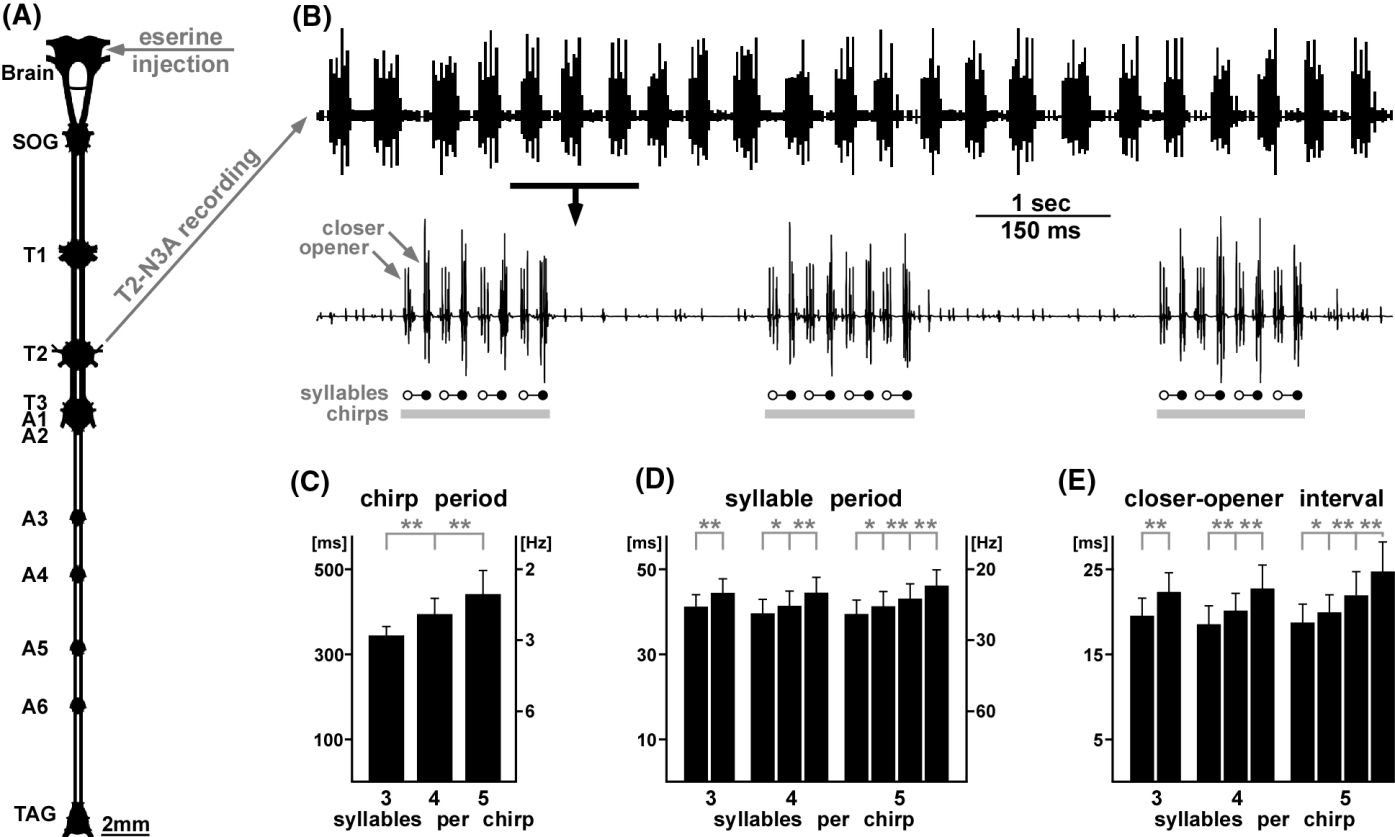
Motor pattern of fictive singing elicited by pharmacological brain stimulation. (A) Ventral view of the cricket central nervous system (CNS) indicating the location of the mesothoracic wing-nerve (T2-N3A) recording and eserine injection into the brain. (B) Chirp rhythm of the fictive singing motor pattern at a low-temporal resolution (top) and the wing-opener and wing-closer motoneuron activity reflecting the syllable pattern at a higher timescale (below). Gray lines indicate chirps and open and filled circles indicate wing-opener and wing-closer motoneuron spike burst, respectively. Chirp periods (C), consecutive syllable periods (D), and consecutive closer-to-opener intervals (E) of 3-, 4-, and 5-syllable chirps (mean ± SD; *N* = 5, *n* = 50; *t*-tests: *P < 0.05, **P < 0.01).

After removing legs and wings, crickets were opened by a dorsal longitudinal incision and pinned out ventral side down onto a plasticine-covered platform. The thoracic and anterior abdominal ganglia were exposed for intracellular recordings, and their peripheral nerves were cut. The head was waxed to a moveable metal support, and a small window was cut in the frontal head capsule to gain access to the brain. Fictive singing was elicited by pressure injection (Pneumatic PicoPump PV820; WPI, Sarasota, FL) of the acetylcholine esterase inhibitor eserine (10^−2^ mol/L in saline; Sigma-Aldrich, St Louis, MO) into the ventral protocerebrum using a blunt glass microcapillary (Fig. 1A; cf. Wenzel and Hedwig 1999; Poulet and Hedwig 2002). Exposed ganglia were continuously rinsed in Ringer's solution for crickets (ionic concentrations in mmol/L: NaCl, 140; KCl, 10; CaCl_2_, 7; NaHCO_3_, 8; MgCl_2_, 1; *N*-trismethyl-2-aminoethanesulfonic acid, 5; d-trehalose dihydrate, 4; pH 7.4).

### Electrophysiological recordings

After severing all thoracic sensory and motor nerves, the motor pattern of fictive singing was recorded extracellularly from the truncated mesothoracic wing nerve 3A (labeled in this article as T2-N3A) using either a double-hook or a suction electrode (Fig. 1B). The signal was amplified with a differential AC amplifier (Model 1700; A-M Systems, Sequim, WA). For intracellular recordings with sharp microelectrodes, the respective ganglion was stabilized between a silver ring and a subjacent silver platform with an embedded optic fiber for brightfield illumination. The microelectrodes were pulled (DMZ-Universal Puller, Zeitz-Instruments, Martinsried, Germany) from borosilicate glass capillaries (GC100F-10, Harvard Apparatus Ltd., Kent, U.K.). Tips were filled with either 4% Lucifer Yellow (Sigma-Aldrich, St Louis, MO) or 5% neurobiotin (Vector Laboratories, Burlingame, CA), and the shaft was backfilled with 1 mol/L lithium chloride or 2 mol/L potassium acetate, respectively. Microelectrodes had final resistances of 90–130 MΩ. Intracellular recorded signals were amplified using a DC amplifier (BA-01X, NPI, Tamm, Germany). All recordings were monitored with an analog oscilloscope (Tektronix 5440) and simultaneously digitized with a 40 kHz sampling rate per channel (Micro1401 mk II, CED, Cambridge, U.K.) for storage on a PC hard drive.

### Structure and terminology of neurons

For anatomical identification, intracellularly recorded neurons were labeled by iontophoretic tracer injection. The ventral nerve cord was subsequently dissected and processed following conventional protocols for Lucifer Yellow (Hedwig 1986) and neurobiotin (Schöneich et al. 2011) labeled whole-mount preparations. Successfully stained neurons were photographed using a digital SLR camera (Canon EOS 350D) attached to a fluorescence microscope (Axiophot, Zeiss, Germany) or scanned with a confocal laser-scanning microscope (Leica SP5, Wetzlar, Germany). The morphology of neurons was graphically reconstructed from the digital image stacks using ImageJ 1.44o (National Institutes of Health, Bethesda, MD). The acronyms given to the identified interneurons reflect morphological and functional features. The first two digits stand for the neuromere housing the cell body. In crickets, the metathoracic ganglion is a fused complex of the metathoracic (T3) and two abdominal (A1 and A2) neuromeres, and the first unfused abdominal ganglion is build by the A3 neuromere. The next two digits indicate by the letter *A*, *D*, or *L* whether it is an ascending, descending, or local interneuron and *O* or *C* stands for rhythmic excitation in phase with the wing-opener or wing-closer motoneurons during singing, respectively.

### Data analysis

Electrophysiological data were analyzed with Spike2 (CED, Cambridge, U.K.) and Neurolab (Knepper and Hedwig 1997). The first spike of the wing-opener and wing-closer motoneuron bursts in nerve T2-N3A was used for interval measurements within the motor pattern and also for delay measurements between interneuron and motor activity. If not stated otherwise, normally distributed data are given as mean ± standard deviation (mean ± SD) and when normality tests failed (Prism 5.0, GraphPad, La Jolla, CA), median and interquartile range (IQR) were used instead. In pooled data sets, each contributing animal is equally represented (*N*, number of animals; *n*, number of stimulations or events).

## Results

### Motor pattern of fictive singing

Male crickets sing by rhythmically opening and closing their front wings and thereby produce a short sound pulse (also called syllable) during each closing movement. In the calling song of *Gryllus bimaculatus*, chirps consisting of 3–5 pulses are perseveringly repeated at a rate of 2–3 Hz, and pulses are generated at a rate of 20–30 Hz within the chirps (Doherty 1985; Verburgt et al. 2011). After severing all sensory and motor nerves of the thoracic ganglia, we elicited fictive singing by pharmacological stimulation of the command neurons in the brain (Fig. 1A; cf. Wenzel and Hedwig 1999). The singing motor pattern was recorded from the left mesothoracic nerve T2-N3A, which contains several axons of wing-opener and wing-closer motoneurons. Thus, the pulse pattern, which constitutes the chirps, is reflected by rhythmically alternating opener- and closer-motoneuron spike bursts in the nerve recordings (Fig. 1B; cf. Poulet and Hedwig 2002). In order to distinguish between the acoustic pulse and the underlying biphasic opener–closer motorcycle, we will refer to the latter as “syllable” as these encompass a silent and sonorous section. To compare the fictive motor pattern with the temporal characteristics of the natural calling song, we quantitatively analyzed the wing-nerve recordings of five males that produced sustained singing episodes with 3-, 4-, and 5-syllable chirps.

In the majority of animals, singing activity started within 20 min after eserine injection and then lasted up to 3 h in some specimen. For episodes of fictive singing with either 3-, 4-, or 5-syllable chirps, the chirp rate decreased significantly with 2.9 ± 0.2, 2.6 ± 0.2, and 2.3 ± 0.3 Hz, respectively (mean ± SD; *N* = 5, *n* = 50; *t*-tests: *P* < 0.001 for each combination; Fig. 1C). This was due to an increase in the chirp duration with each additional syllable generated (108 ± 7, 148 ± 10, and 192 ± 12 msec for 3-, 4-, and 5-syllable chirps, respectively; *N* = 5, *n* = 50; *t*-tests: *P* < 0.001 for each combination). In contrast, regardless of the chirp duration, the chirp intervals ranged between 210 and 256 msec (IQR; median = 233 msec; *N* = 5, *n* = 150).

When pooled over the five animals, the mean syllable rate was 23.8 ± 2.2 Hz (mean ± SD; *N* = 5, *n* = 450). From the beginning to the end of a chirp, however, consecutive syllables became longer, resulting in a gradual decrease in the instantaneous syllable rate (Fig. 1D). For 5-syllable chirps, the consecutive syllable repetition rates were 25.5 ± 2.3, 24.3 ± 1.6, 23.3 ± 1.6, and 21.8 ± 1.7 Hz; for 4-syllable chirps 25.3 ± 2.2, 24.3 ± 2.1, and 22.6 ± 2.0 Hz; and for 3-syllable chirps 24.3 ± 1.8 and 22.6 ± 1.8 Hz (mean ± SD; *N* = 5, *n* = 50). The mean syllable rate of chirps was very consistent for each individual animal regardless of the syllable number, but between males it varied significantly in the range of 21–26 Hz (*t*-test: *P* < 0.0001 for seven of the 10 possible combinations between five animals; *n* = 90 each).

During fictive singing, an opener-to-closer interval of 21.5 ± 2.1 msec (*N* = 5, *n* = 600) and subsequent closer-to-opener interval of 21.0 ± 3.2 msec (*N* = 5, *n* = 450) were generated (lower trace in Fig. 1B). The average opener–closer interval differed significantly in a range from 20 ± 2 to 24 ± 1 msec between individual males (*t*-test animal min vs. animal max: *P* < 0.0001; *n* = 120 each), but was highly consistent for each animal regardless of instantaneous chirp or syllable rate and the number of syllables per chirp. Although with 21.2 msec (mean ± SD; *N* = 5, *n* = 150), the opener–closer interval of the first syllable in a chirp was in average by 0.4 msec shorter than the following (*t*-test first vs. second and first vs. third syllable: *P* < 0.01; second vs. third: *P* > 0.8; *N* = 5, *n* = 150 each), the progressively decreasing syllable rate within chirps (Fig. 1D) resulted mainly from the gradual lengthening of the closer–opener interval (Fig. 1E). For 5-syllable chirps, the closer–opener intervals of the consecutive syllables were 18.8 ± 2.2, 20.0 ± 2.1, 22.0 ± 2.8, and 24.8 ± 3.5 msec; for 4-syllable chirps, 18.6 ± 2.2, 20.2 ± 2.1, and 22.8 ± 2.7 msec; and for 3-syllable chirps, 19.6 ± 2.1 and 22.4 ± 2.3 msec (mean ± SD; *N* = 5, *n* = 50).

In summary, our quantitative data analysis demonstrates that the motor pattern of fictive singing is remarkably rigid and robust. Even though it lacks in any sensory feedback, it closely reflects the temporal pattern of the natural calling song in all details (cf. Kutsch 1969; Hennig 1989).

### Cellular analysis of the singing network

Interneurons of the singing pattern generating network were intracellularly recorded and stained in the metathoracic ganglion (encompassing the T3, A1, and A2 neuromeres) and first unfused abdominal ganglion (A3 neuromere). Recent experiments had revealed that these two ganglia house the singing-pattern generator (Schöneich and Hedwig 2011). To test whether a recorded interneuron was part of the singing CPG, we modulated its spike activity by intracellular current injection and analyzed its impact on the ongoing motor pattern. An interneuron was considered a component of the singing CPG if its rhythmic activity strictly preceded the opener- or closer-motoneuron bursts and if transient perturbations of its activity reset or considerably altered the motor pattern. To quantitatively analyze the timing of interneuron activity with respect to the syllable rhythm, we used the spike burst onset of wing-opener and wing-closer motoneurons as temporal reference. The anatomical and physiological characteristics of individual singing interneurons are described in the following paragraphs.

### Ascending opener-interneuron A3-AO

**Figure 2:**
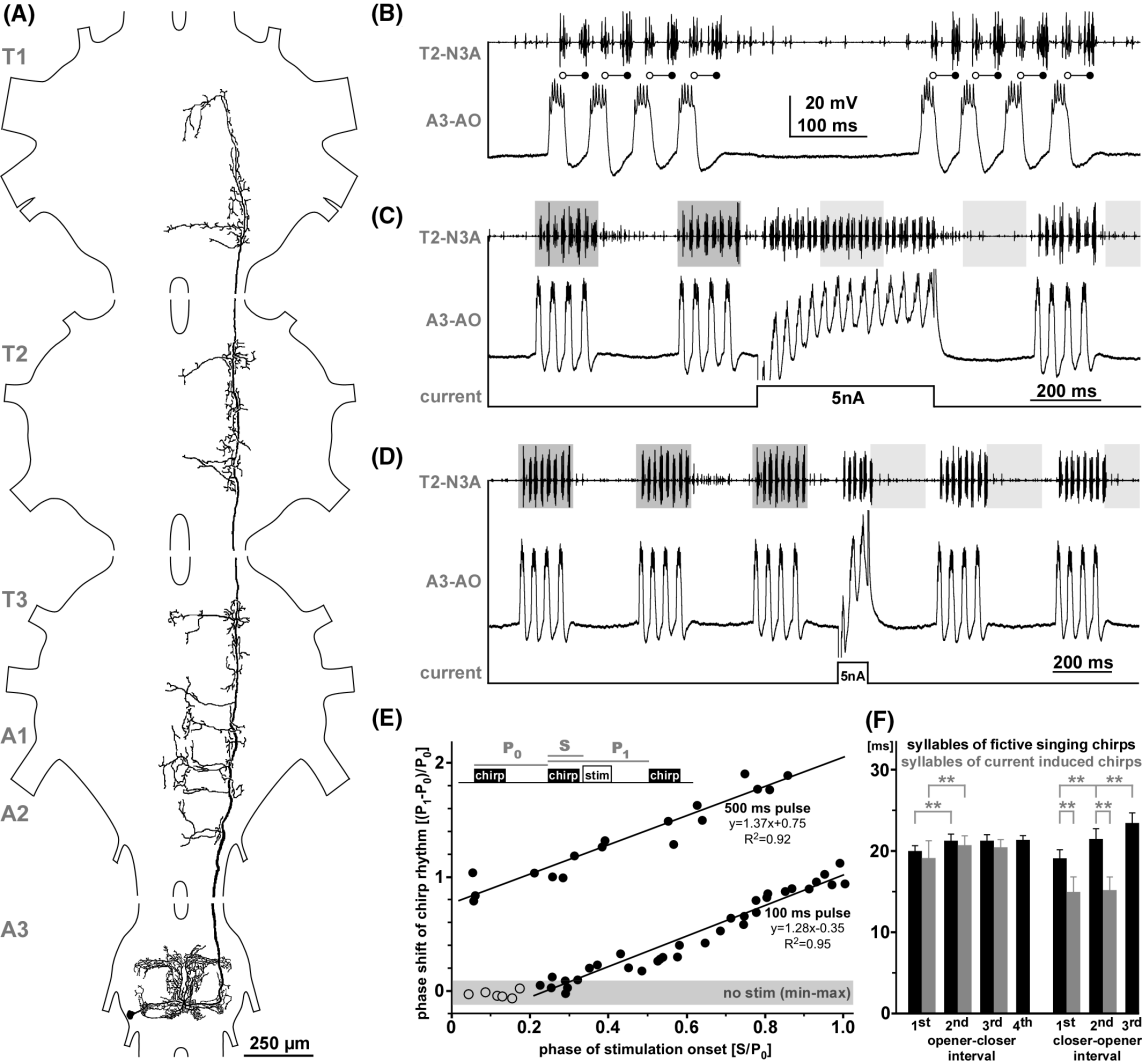
Structure and activity of the abdominal ascending opener-interneuron A3-AO. (A) Morphology of A3-AO with cell body and dendrites in A3 and axonal projections in thoracic ganglia (ventral view). (B–D) Singing motor pattern (top trace) and activity of A3-AO (lower trace). (B) During fictive singing A3-AO depolarized and spiked in phase with wing-opener activity (open circles) and hyperpolarized in phase with the wing-closer activity (filled circles). (C) Intracellular current injection of 5 nA for 500 msec (bottom trace) elicited rhythmic A3-AO activity causing a long 14-syllable chirp. (D) Intracellular injection of 5 nA for 100 msec (bottom trace) elicited an additional 3-syllable chirp. (C) and (D) Reset of the chirp rhythm by the current injection elicited chirps. Gray boxes indicate the chirp rhythm before stimulation and light-gray boxes continue this rhythm. (E) Phase–response diagram for current pulses of 100 msec (*N* = 3, *n* = 34; lower trend line) and 500 msec duration (*N* = 3, *n* = 17; upper trend line) shows that the shift of the chirp rhythm depended linearly on stimulus phase and current pulse duration. Open circles (*N* = 3, *n* = 6) represent 100 msec current pulses falling entirely within a chirp. The gray area indicates the variation in the chirp period before stimulation (min–max range; *N* = 3, *n* = 60). (F) The opener–closer interval of the consecutive syllables did not differ between 3-syllable chirps induced by current injection (gray bars) and 4-syllable chirps of fictive singing (black bars). The closer–opener intervals, however, were significantly reduced in the current-induced chirps and did not show the typical increase in duration for consecutive syllables (mean ± SD; *N* = 3, *n* = 45; *t*-tests: *P < 0.05, **P < 0.01).

**Figure 3:**
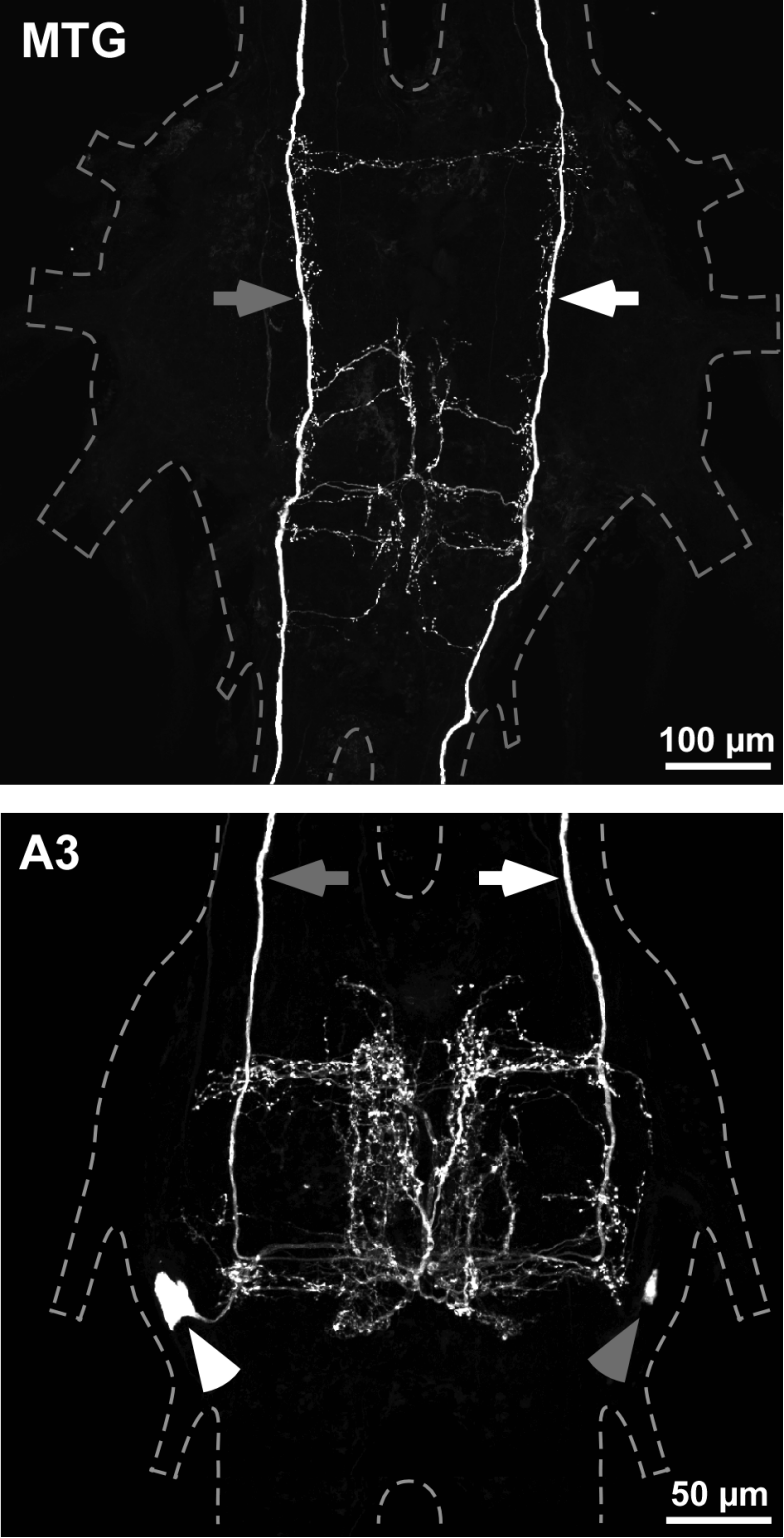
Dye coupling of the two A3-AO sibling neurons with neurobiotin. Extended focus views (maximum intensity projections of confocal image stacks) showing the fluorescence-labeled (neurobiotin-avidinCy3) arborizations of the two bilateral symmetrical A3-AO neurons in the first unfused abdominal ganglion (A3; lower picture) and metathoracic ganglion complex (MTG; upper picture) after intracellular injection of neurobiotin in one of them. Stippled lines indicate the ganglion outline; arrowheads mark the somata and arrows the ascending axons.

In the abdominal ganglion A3, we identified the interneuron A3-AO that discharged in phase with the syllable rhythm. This neuron was intracellularly recorded in 12 animals and subsequently stained with either Lucifer Yellow (*N* = 5) or neurobiotin (*N* = 3); it was described as A3-IN in a preliminary report by Schöneich and Hedwig (2011). The soma of A3-AO was located on the lateral margin of A3, the neurite crossed the ganglion midline close to the dorsal surface and its contralaterally ascending axon projected up to the prothoracic ganglion (Fig. 2A). At both sides of A3, the primary neurite gave off a large anterior and a smaller posterior dendrite. The ipsilateral and contralateral dendrites branched out bilateral symmetrically in the very dorsal neuropile of A3. In each neuromere of the three thoracic ganglia, one prominent anterior branch and one or two posterior branches arose from the main axon and projected dorsally toward the midline of the respective ganglion. A3-AO occurred as a bilateral pair of sibling neurons, and in all three neurobiotin-labeled specimen, the mirror-image sibling neuron was clearly stained as well (Fig. 3). Such dye coupling may indicate electrical coupling between the left and right A3-AO interneuron via gap junctions (cf. Ewadinger et al. 1994; Fan et al. 2005; Anava et al. 2009).

During fictive singing, the membrane potential of A3-AO, as recorded in its main dendrite, oscillated with the syllable pattern (Fig. 2B). In the opener phase of each syllable, it depolarized by 20–25 mV, and in the closer phase, it hyperpolarized 5–10 mV beneath resting potential. The depolarization preceding the first syllable of a chirp was up to 3.5 mV higher than the following. Every depolarization gave rise to a volley of 4–6 action potentials with a spike frequency of up to 380 Hz during the first syllable and 360 Hz during the following syllables of a chirp. The spike activity in A3-AO preceded the next opener burst in the wing nerve by 10.1 ± 0.8 msec (mean ± SD; *N* = 10) and the following closer burst by 29.2 ± 2.2 msec (mean ± SD; *N* = 10). Therefore, we refer to A3-AO as an opener interneuron. Interestingly, we never observed any synaptic inputs or spike activity in the A3-AO dendrite before and after singing episodes or during chirp intervals.

When a constant depolarizing current of 5 nA was injected into the dendrite of A3-AO, the interneuron responded with consistently repeated depolarization–hyperpolarization oscillations of its membrane potential generating a burst of 2–6 action potentials during each depolarization. This rhythmic interneuron activity reliably elicited alternating opener–closer motoneuron activity reflecting the normal syllable pattern in the ipsi- and contralateral wing nerves. Activation of a single A3-AO interneuron is therefore sufficient to continuously drive the motor pattern of the syllable rhythm. Current pulses of 500 msec elicited long chirps with 14–15 syllables (Fig. 2C) and pulses of 200 msec strictly elicited 6-syllable chirps. Injecting +5 nA for just 100 msec during the chirp interval caused strictly three additional depolarization–hyperpolarization cycles and the motor pattern of an additional 3-syllable chirp (Fig. 2D). Short current pulses (+5 nA; 10–20 msec), which fell entirely within a chirp, did not change the singing pattern. When injected during the chirp intervals, however, they reliably triggered a single membrane potential oscillation-cycle with at least two action potentials that strictly elicited the motor pattern of a single syllable.

Each additional chirp evoked by depolarizing current injection to A3-AO reliably reset the chirp rhythm of the singing activity (Fig. 2C and D). After the end of the stimulus, the subsequent chirp started with a delay of 230 ± 34 msec (*N* = 3, *n* = 51), which closely matched the duration of the normal chirp intervals (229 ± 20 msec; *N* = 3, *n* = 60) before current injection. Injection of 100 msec and 500 msec current pulses at different moments of the chirp cycle revealed a linear correlation between the stimulation phase and the resulting phase shift of the chirp rhythm (Fig. 2E). Plotted as a phase–response curve (Pinsker 1977), the data for 100 and 500 msec current pulses were closely fitted by the linear regression functions *y* = 1.28 × −0.35 (*R*^2^ = 0.95; *N* = 3, *n* = 34) and *y* = 1.37 × +0.75 (*R*^2^ = 0.92; *N* = 3, *n* = 17), respectively. The trend lines of the two data sets are vertically shifted by 1.1 chirp cycles (mean chirp cycle: 364 ± 43 msec; *N* = 3, *n* = 120), which precisely reflect the difference of 400 msec in stimulus duration. As A3-AO activation is sufficient to drive the syllable motor pattern and also reliably reset the chirp rhythm, this interneuron is clearly a pivotal element of the cricket singing CPG.

There was no significant difference between the average opener–closer intervals of fictive singing chirps (21 ± 1 msec; *N* = 3, *n* = 90) and chirps induced by current injection in the A3-AO dendrite (20 ± 2 msec; *N* = 3, *n* = 90). Just as in the fictive singing pattern, the opener–closer interval of the first syllable in the current-induced chirps was slightly shorter compared with the following (*t*-test first vs. second and first vs. third syllable: *P* < 0.01; second vs. third: *P* > 0.5; *N* = 3, *n* = 21 each). The closer–opener intervals, however, were significantly reduced (*t*-test: *P* < 0.0001; *N* = 3, *n* = 45) in current-induced chirps (mean ± SD: 15 ± 2 msec) compared with fictive singing (mean ± SD: 21 ± 2 msec) and did not show the successive increase as in natural chirps (Fig. 2F).

**Figure 4:**
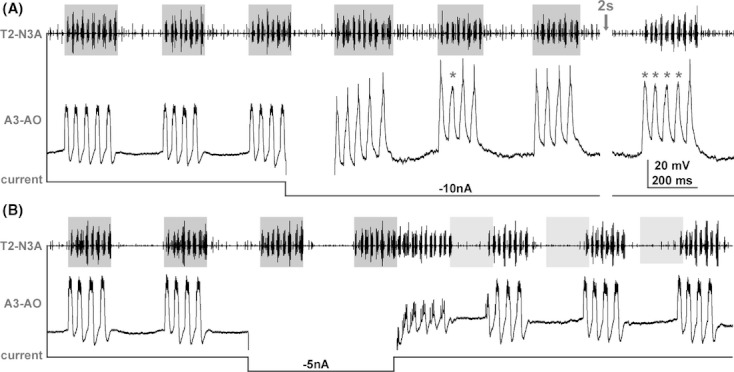
Effect of A3-AO hyperpolarization on fictive singing. (A) Sustained current injection with −10 nA reduced and suppressed (asterisks) the spike activity of A3-AO without influencing the ongoing singing motor pattern; arrow indicates a 2-sec gap of the continuous recording. (B) Transient hyperpolarization of A3-AO with −5 nA was followed by rhythmic bursting of A3-AO, which elicited additional syllables in the motor pattern and reset the chirp rhythm. Gray boxes indicate the chirp rhythm before stimulation and light-gray boxes continue this rhythm.

Sustained hyperpolarizing current injection was used to test if spike activity in both A3-AO sibling neurons is necessary to maintain fictive singing. Within 15–20 sec of injecting a constant −10 nA current in the dendrite of one A3-AO interneuron, fictive singing stopped and recurred not until 5–10 sec after the current injection. During the first seconds of hyperpolarization, the rhythmic spike activity in A3-AO was drastically reduced and even completely abolished for some individual syllables (Fig. 4A). This reduced activity in one A3-AO neuron, however, did not affect the ongoing singing motor activity, indicating that the single A3-AO interneuron is not necessary for the cycle-by-cycle generation of the singing motor pattern and the spike activity of the contralateral A3-AO neuron was presumably sufficient to transiently maintain the motor output. Interestingly, short hyperpolarizing current pulses (−5 nA; 100–1000 msec duration) in the A3-AO dendrite were immediately followed by additional membrane potential oscillations in this neuron (Fig. 4B). Although the depolarization amplitudes of the post-hyperpolarization response were considerably smaller (2–6 mV) than the opener-phase depolarizations during fictive singing (20–25 mV), A3-AO generated a burst of 3–5 action potentials during each poststimulus depolarization, which elicited a corresponding sequence of syllables in the motor pattern that reset the ongoing chirp rhythm (Fig. 4B).

**Figure 5:**
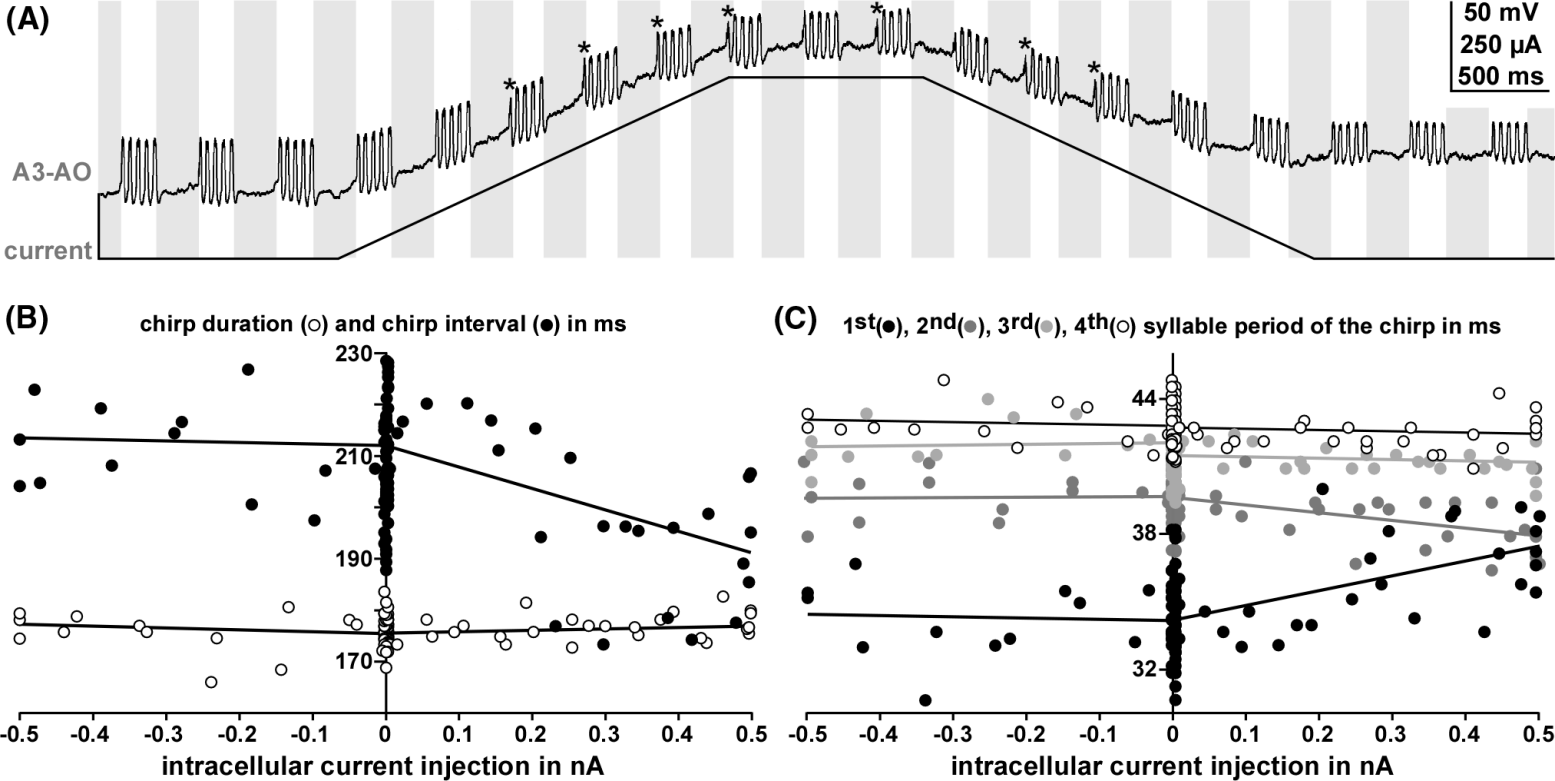
Modulation of the singing pattern by subthreshold membrane potential shifts in A3-AO. (A) Ramp-like current injection with up to 0.5 nA amplitude in the dendrite of A3-AO did not influence the number of syllables per chirp but reduced the chirp intervals. Gray bars indicate the average chirp interval duration before current injection. Chirps starting considerably earlier are marked with asterisks. (B) The stimulus–response plot shows that depolarizing current injection did not significantly alter the chirp duration (open circles; *R*^2^ = 0.02 for linear fit) but considerably reduced the duration of the chirp intervals (filled circles; *R*^2^ = 0.2 for linear fit). Hyperpolarizing current injection did influence neither chirp duration nor chirp interval (linear fits: *R*^2^ = 0.02 and *R*^2^ = 0.05, respectively). (C) With increasing depolarization, the first syllable period in the chirps (black circles) was progressively lengthened (linear fit: *R*^2^ = 0.3) and the second syllable period (dark gray circles) shortened (linear fit: *R*^2^ = 0.2). The third and fourth syllable periods (light gray and open circles, respectively) remained unchanged (*R*^2^ < 0.02 for both linear fits). Hyperpolarization had no influence on the syllable periods (*R*^2^ < 0.02 for each linear fit).

The intracellular current injection experiments demonstrated the importance of A3-AO spike activity for the singing pattern generation. By gradual manipulation of its membrane potential, we asked if also subthreshold stimulation would modulate the singing activity. Ramp-like depolarizing and hyperpolarizing current with maximum amplitudes of only +0.5 nA and −0.5 nA was injected into the dendrite of A3-AO. This gently shifted the membrane potential of the neuron without changing the number of syllables per chirp (Fig. 5A) or even the spike activity underlying each syllable (A3-AO spikes per syllable: 0 nA, 4.7 ± 0.6; −0.5 nA, 4.7 ± 0.5; +0.5 nA, 4.6 ± 0.7; mean ± SD; *N* = 1, *n* = 25 each). The low-amplitude current injection did not influence the average chirp duration, which remained 176 ± 5 msec throughout the experiment. The duration of the chirp intervals (212 ± 18 msec; mean ± SD before current injection), however, progressively decreased with increasing depolarization (199 ± 15 msec for 0.1–0.3 nA; 192 ± 13 msec for 0.3–0.5 nA), whereas moderate hyperpolarization had no effect (Fig. 5B). Similarly, the initial syllable periods within the chirps were modulated by moderate depolarization but not by hyperpolarization (Fig. 5C). With increasing depolarization, the first syllable period in a chirp was lengthened by up to 4 msec, the second syllable period was shortened by up to 2 msec, and the following syllable periods did not change. These subtle modulations of the singing motor pattern indicate that the temporal structure of the motor output does not only depend on the spike activity but also on graded changes in the membrane potential of A3-AO.

### Descending opener-interneuron T3-DO

**Figure 6:**
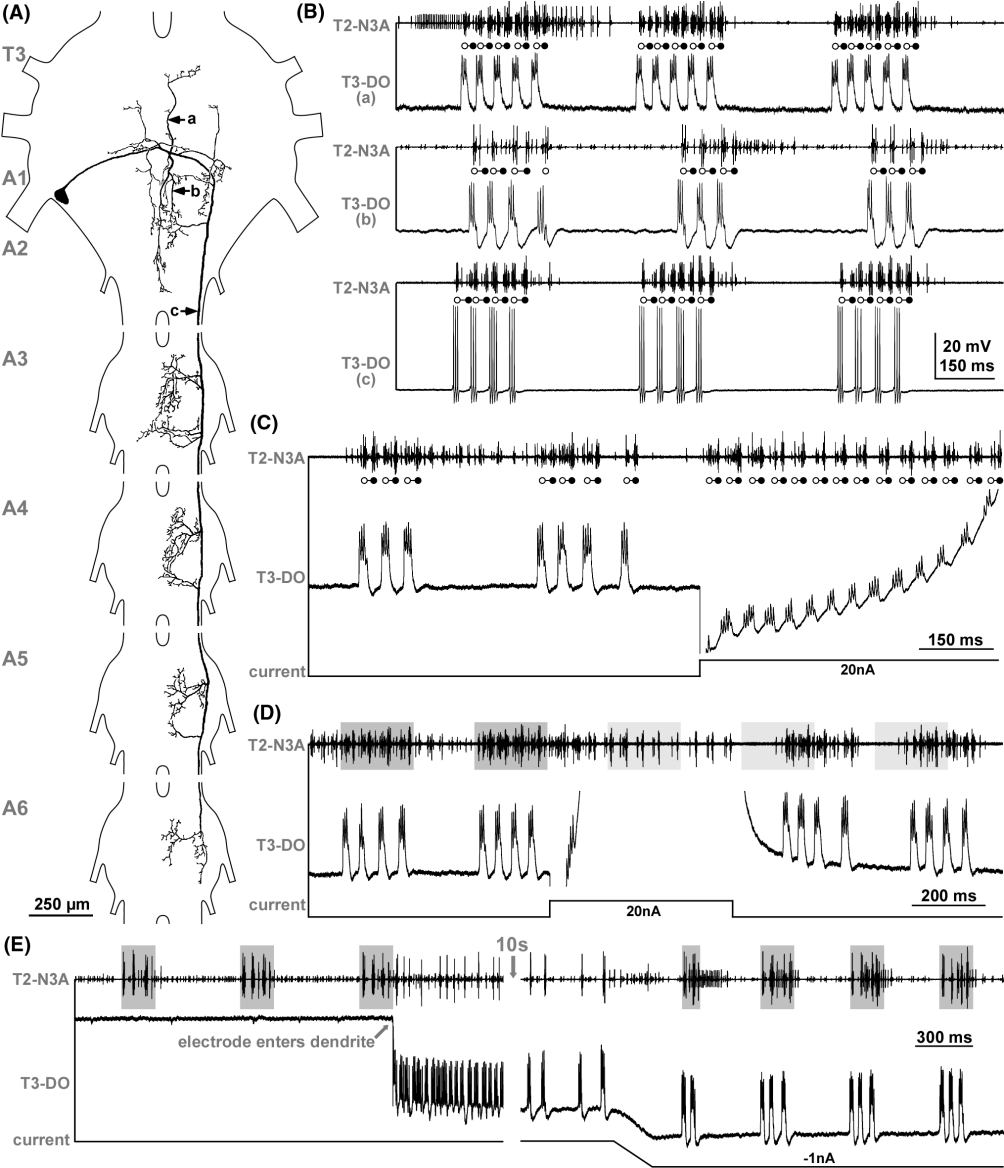
Structure and activity of the thoracic descending opener-interneuron T3-DO. (A) Cell body, neurite, and dendrites in the metathoracic ganglion complex and axonal branches in abdominal ganglia A1–A6 (ventral view). (B–E) Singing motor pattern (top traces) with wing-opener (open circles) and wing-closer (filled circles) activity and the activity of T3-DO (lower traces). (B) Recordings from different T3-DO branches during fictive singing as indicated by arrows in (A). Recordings from the anterior dendrite (a) demonstrate membrane depolarization and generation of 3–4 spikes in the opener phase; posterior dendrite recordings (b) also reveal a hyperpolarization in the closer phase; axonal recordings (c) show only spike activity. (C) Sustained depolarization with 20 nA evoked rhythmic spike activity in T3-DO and elicited ongoing cycles of opener- and closer-motoneuron bursts. (D) A 500 msec pulse of 20 nA evoked the motor pattern of 11 additional syllables, which reset the ongoing chirp rhythm; gray boxes indicate the chirp rhythm before stimulation and light-gray boxes continue this rhythm. (E) Microelectrode penetration caused bursting activity in T3-DO, which immediately stopped the singing activity. During recovery, the bursting activity of the neuron gradually slowed down. On hyperpolarizing the membrane with −1 nA, the interneuron discharged again in the singing pattern, and at the same time, the normal motor output of fictive singing was reconstituted (gray boxes indicate chirps).

Systematic probing the metathoracic neuromere with microelectrodes provided little evidence for the presence of singing interneurons. Only close to the border toward the A1 neuromere could we identify an interneuron with a contralateral descending axon that discharged in phase with the singing rhythm. The neuron was intracellularly recorded in 17 animals and subsequently stained with either Lucifer Yellow (*N* = 7) or neurobiotin (*N* = 3). The cell body of T3-DO was located on the lateral margin of the metathoracic ganglion just posterior to the root of nerve 5 (Fig. 6A). From there, the primary neurite ran dorsally along the border between the metathoracic and first abdominal neuromere toward the contralateral side. Near the midline of the ganglion, one prominent posterior and three anterior dendrites arose from the primary neurite. In all stained specimens, the most conspicuous feature of this neuron was the posteriorly projecting dendrite that branched along the dorsal midline of the two abdominal neuromeres (A1 and A2). The arborization patterns of the much thinner anterior dendrites varied considerably between animals. In the metathoracic ganglion, the contralateral descending axon had one medially projecting side branch in A1 and one in A2, which both ramified dorsally near the midline of the ganglion. In the unfused abdominal ganglia A3–A6, anterior and posterior axonal side branches projected in a similar way toward the dorsal midline neuropile, while the diameter of the descending axon decreased progressively and the axonal arborizations became less extensive from ganglion to ganglion. The axon of T3-DO typically terminated in A6, but in two animals, it descended as a very thin fiber toward the terminal ganglion.

Interneuron T3-DO fired bursts of 3–4 action potentials in phase with the syllable rhythm of fictive singing. Spike bursts started strictly 7.0 ± 0.8 msec (mean ± SD; *N* = 10) before the opener-motoneuron activity and 26.9 ± 3.2 msec (mean ± SD; *N* = 10) before the closer-motoneuron spike bursts (Fig. 6B), characterizing it as an opener interneuron. Recordings from the posterior dendrite revealed that the membrane potential clearly oscillated in phase with the syllable rhythm. In the opener phase, the dendrite depolarized by 4–6 mV, and in the closer phase, it hyperpolarized 7–8 mV below the resting potential. In recordings from the anterior main dendrite, the depolarization in the opener phase reached up to 14 mV and the hyperpolarization in the closer phase was hardly apparent. This indicates that the closer-phase inhibition occurs predominantly at the posterior dendrite, whereas the opener-phase depolarizations are more pronounced in the anterior dendrites. Recordings from the axon revealed just the rhythmic spike activity of the neuron, which reached 160–200 Hz during the opener phases. In the dendrite of T3-DO, the membrane depolarization coupled to the first syllable of a chirp was slightly less pronounced as compared with the following syllables. Like in the opener-interneuron A3-AO, also in T3-DO we never observed spike activity or any synaptic inputs before or after singing episodes or during the chirp intervals.

When we tried to disturb the ongoing singing activity by electrical stimulation of T3-DO, it became apparent that its main dendrite required exceptionally strong depolarizing current pulses of 10–20 nA to evoke additional spike activity. However, when effectively stimulated, the neuron generated rhythmic membrane potential oscillations for the duration of the current injection (Fig. 6C). During each of the depolarization phases, the neuron produced a burst of 3–5 action potentials, which elicited a cycle of opener–closer motoneuron activity reflecting the syllable pattern of fictive singing. Current pulses of 20 nA amplitude and just 20 msec duration were sufficient to evoke a single syllable, current pulses of 500 msec released 10–12 syllables, and sustained current injection for 1 sec caused a continuous train of 20–25 syllables. Similar to the reset effect of A3-AO, the additional syllables elicited by current injection in T3-DO reset the chirp rhythm in a way that after stimulation the next chirp cycle always started with a normal chirp interval of 180–250 msec (Fig. 6D). The current injection experiments demonstrated that depolarizing T3-DO beyond its spiking threshold is sufficient to drive the syllable motor pattern of fictive singing and reset the chirp rhythm.

During fictive singing even strong hyperpolarizing current injection of −20 nA in the main dendrite of T3-DO did not evidently reduce the spike activity of the interneuron, although it clearly reversed the polarity of the inhibitory postsynaptic potentials (IPSPs) in the closer phase. However, elevated T3-DO spike activity of about 100 action potentials per second, which occasionally occurred when the recording electrode entered the dendrite of the neuron, stopped fictive singing immediately (Fig. 6E). Interestingly, the high-frequency spike activity of T3-DO was still organized in bursts of 3–4 spikes, which were separated by brief phases of hyperpolarization. Moreover, the concomitantly occurring large-amplitude motoneuron spikes in the wing nerve were strictly latency coupled to the preceding interneuron burst (latency: 24 ± 2 msec; mean ± SD; *N* = 1, *n* = 50). Within a few seconds of recovery after microelectrode penetration, the mean burst frequency of T3-DO gradually decreased to about 10 Hz and the inhibition following each burst became more and more prominent. On subsequent intracellular injection of hyperpolarizing current (−1 nA), the spike bursts of T3-DO became grouped into the normal chirp pattern, and at the same time, the motor output of fictive singing was instantaneously reconstituted (Fig. 6E).

### Ascending opener-interneuron A1-AO

**Figure 7:**
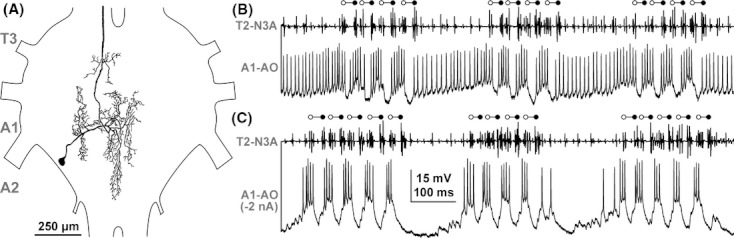
Structure and activity of the ascending opener-interneuron A1-AO. (A) Cell body position and dendrites of A1-AO in the fused abdominal–metathoracic ganglion complex (ventral view); the axon ascends toward the mesothoracic ganglion. (B) and (C) Singing motor pattern (top trace) with wing-opener (open circles) and wing-closer activity (filled circles) and A1-AO activity (lower trace). During fictive singing A1-AO depolarized and generated 3–6 spikes in the opener phase and it hyperpolarized in phase with wing-closer activity. During the chirp intervals the neuron fired tonically. (C) Hyperpolarizing current injection of −2 nA in the dendrite of A1-AO reduced the rhythmic spike activity and suppressed tonic spiking during the chirp intervals but did not alter the singing motor pattern.

We also identified an ascending interneuron in the metathoracic ganglion complex that spiked rhythmically in phase with the wing-opener activity and that was inhibited in phase with the wing-closer motoneurons. Its soma was located at the lateral margin of the first fused abdominal neuromere A1, from where its primary neurite ran dorsally toward the posterior border of the metathoracic neuromere (Fig. 7A). Forming a loop near the ganglion midline, the main neurite sharply bent anteriorly and the ascending axon projected through the ipsilateral connective toward the mesothoracic ganglion. Before leaving the ganglion, the axon gave off a side branch that ramified dorsally in the anterior metathoracic neuromere. Arising from the neurite, the main dendrite of A1-AO formed a dense meshwork of fine branches projecting anteriorly and posteriorly along the dorsal midline of the neuromeres A1 and A2.

During fictive singing, the membrane potential of A1-AO depolarized by 4–8 mV in each opener phase and hyperpolarized by 4–5 mV in phase with the closer-motoneuron activity (Fig. 7B). Every depolarization gave rise to a burst of 3–6 action potentials with an instantaneous spike frequency of 140–180 Hz. During each syllable, A1-AO fired its first spike 7.5 ± 1.1 msec (mean ± SD; *N* = 1, *n* = 50) before the first spike of the wing-opener motoneuron activity and 31.2 ± 1.2 msec (mean ± SD; *N* = 1, *n* = 50) before the first spike of the wing-closer activity. During the chirp intervals, the neuron spiked tonically at a rate of 100–120 Hz. This tonic background activity might result from a slightly elevated membrane potential due to the microelectrode penetration. Constant hyperpolarizing current injection in the dendrite of A1-AO completely prevented tonic spiking during the chirp intervals and also reduced the rhythmic spike activity during chirps (Fig. 7C). The spike activity reduction in A1-AO did not affect the singing motor pattern and neither strong depolarizing nor hyperpolarizing current pulses reset the chirp rhythm of fictive singing.

### Closer interneurons

While recording in the abdominal neuromeres, we encountered considerably more often opener interneurons than closer interneurons. Nevertheless, in 12 crickets, we recorded interneurons whose rhythmic spike activity was strictly coupled to the closer phase of fictive singing. Here, we describe one morphologically identified closer interneuron and report on functional significant data from two closer interneurons, which were not labeled.

**Figure 8:**
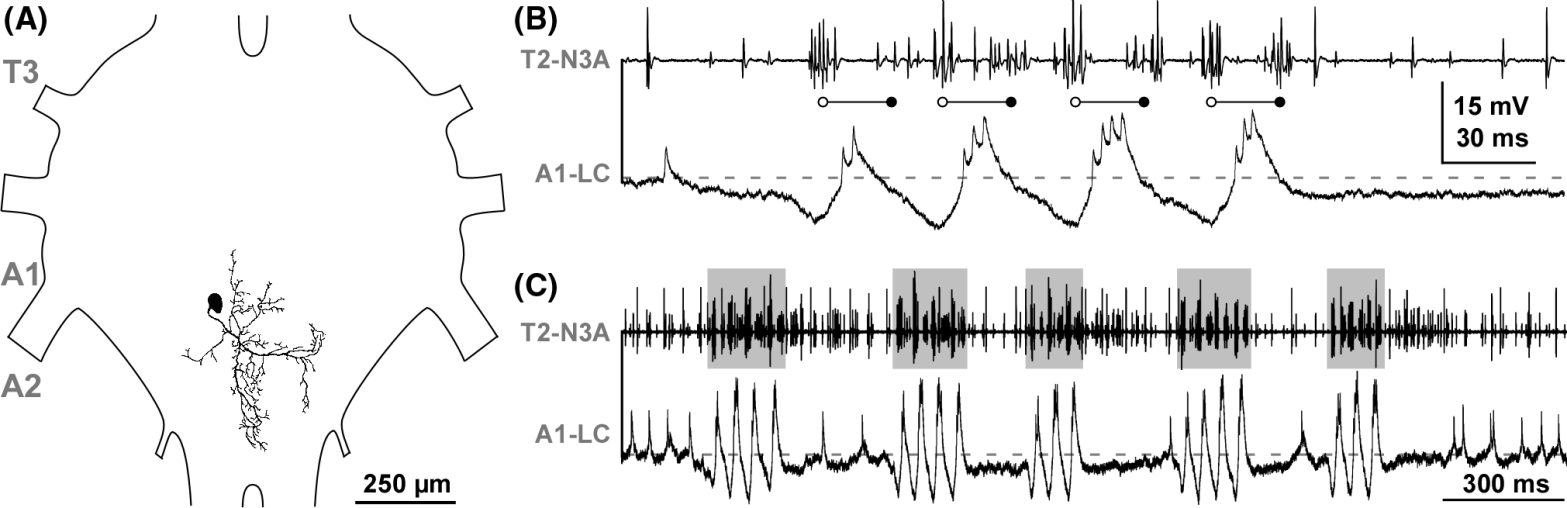
Structure and activity of the local closer-interneuron A1-LC. (A) Morphology of A1-LC in the fused abdominal neuromeres of the metathoracic ganglion (ventral view). (B) and (C) Singing motor activity (top trace) and intracellular recordings of A1-LC (lower trace) with a dashed line marking the resting potential. (B) A 4-syllable chirp at high-temporal resolution (open and filled circles indicate wing-opener and wing-closer bursts, respectively). During fictive singing A1-LC hyperpolarizes in phase with wing-opener activity and depolarizes generating 2–4 spikes in phase with wing-closer activity. (C) During singing episodes (gray boxes indicate chirps), the membrane potential of A1-LC dropped in the chirp breaks slightly below the resting potential and the background spike activity was largely reduced.

A1-LC is a local closer interneuron in the A1 neuromere of the metathoracic ganglion (Fig. 8A). Its dorsal cell body was located near the ganglion midline and its primary neurite projected toward the contralateral ganglion side. An anterior and a posterior dendritic main branch arose from the primary neurite at the midline of the ganglion and ramified along the dorsal midline of A1 and A2 where they spatially overlapped with the posterior dendrite of T3-DO and axonal branches of A3-AO and T3-DO. During fictive singing, A1-LC was depolarized and generated 2–4 action potentials in each wing-closer phase and was inhibited during the wing-opener phase (Fig. 8B). For each syllable, the neuron fired its first spike 11.4 ± 1.5 msec (mean ± SD; *N* = 1, *n* = 20) after the first wing-opener motoneuron spike and 10.2 ± 1.1 msec (mean ± SD; *N* = 1, *n* = 20) before the first spike of the wing-closer burst. During the chirp intervals, the membrane potential of A1-LC was up to 3 mV below the resting potential, which drastically reduced its spontaneous spike activity from 23 Hz before and after singing episodes to a mean spike activity of 8 Hz during chirp intervals.

**Figure 9:**
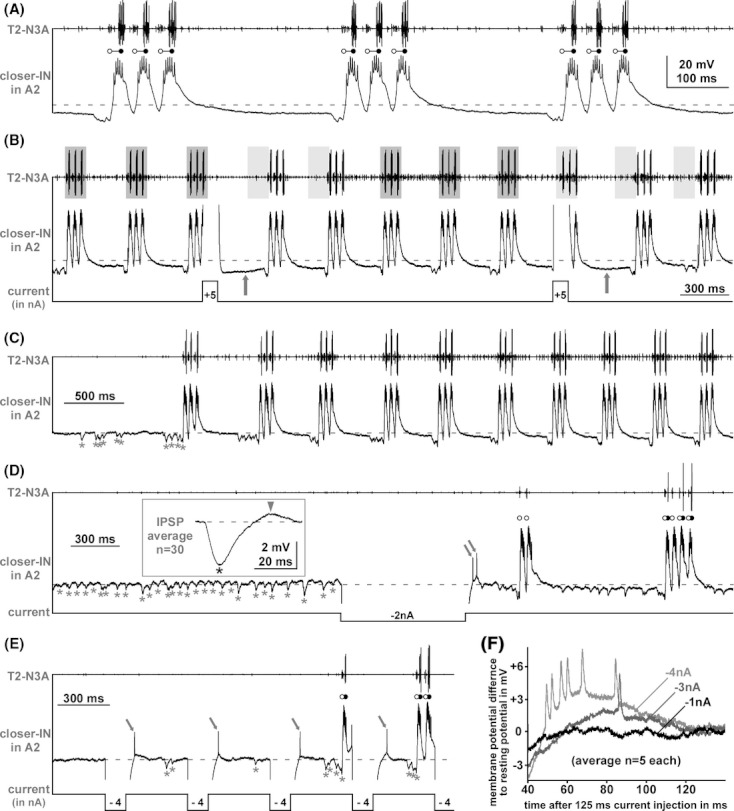
Postinhibitory rebound activation of a morphologically nonidentified closer interneuron recorded in A2. (A–E) Singing motor activity (top trace) and intracellular dendritic recordings of the interneuron (lower trace). A dashed line marks the resting potential. (A) During fictive singing the interneuron depolarized and spiked in phase with wing-closer activity (filled circles) and hyperpolarized in phase with wing-opener activity (open circles). The neuron was hyperpolarized during the chirp intervals and received additional inhibition at the beginning of the next chirp. (B) Depolarizing current pulses (5 nA/100 msec) reset the chirp rhythm but did not elicit additional motor activity; gray boxes indicate an unchanged chirp pattern. The membrane potential significantly dropped during the prolonged chirp interval following stimulation (arrows). (C) Before a singing episode the interneuron received volleys of IPSPs (marked with asterisks). During the first seconds of singing the membrane potential progressively hyperpolarized and the IPSPs became confined to the beginning of chirps. (D) After a singing episode the interneuron received trains of IPSPs; each followed by a slight depolarization. Averaging 30 IPSPs (inset) revealed postinhibitory rebound depolarization. Also a hyperpolarizing current pulse of −2 nA/700 msec elicited a transient membrane depolarization and spikes (arrows) followed by a short episode of singing. (E) Repeated (2 Hz) hyperpolarizing current pulses of −4 nA/125 msec were each followed by a short depolarization with spike response (arrows) and after a delay of 200–300 msec by IPSPs (asterisks); finally brief episodes of singing activity were released. (F) Averaged (*n* = 5) responses of the neuron's membrane potential after −1, −3, and −4 nA hyperpolarizing 125 msec current pulses reveal different degrees of postinhibitory rebound depolarization depending on the stimulus amplitude.

In the A2 neuromere, we recorded a morphologically unidentified closer interneuron that received conspicuous inhibition at the beginning of each chirp and indicated postinhibitory rebound as a presumable mechanism contributing to singing pattern generation. During fictive singing, this closer neuron was inhibited in each opener phase and depolarized by 20–25 mV in the closer phase (Fig. 9A). Every depolarization gave rise to a burst of 5–6 action potentials with a spike frequency of 250–300 Hz. During each syllable, the neuron fired its first spike 12.0 ± 2.3 msec (mean ± SD; *N* = 1, *n* = 50) after the start of the wing-opener motoneuron burst and 8.0 ± 0.4 msec (mean ± SD; *N* = 1, *n* = 50) before the first spike of the wing-closer burst. Injection of depolarizing current pulses (+5 nA; 100 msec) reset the ongoing chirp rhythm similar to A3-AO and T3-DO, but in contrast to the reset effect of the opener interneurons, electrical stimulation of this closer neuron did not elicit additional singing motor activity (Fig. 9B). Interestingly, during the chirp intervals following the current pulses (arrows in Fig. 9B), the membrane potential was about 3 mV lower as during the preceding and following chirp intervals. Before the start of each singing episode, this closer interneuron received several volleys of 4–6 individual IPSPs at a time (Fig. 9C). After singing started, similar IPSP volleys preceded each chirp, and while the chirp rate increased during the first seconds of a singing episode, the individual IPSPs within each volley started to coincide more and more. When singing was in full swing (Fig. 9A), the membrane potential during the chirp intervals was up to 5 mV below the resting potential, and in addition, every chirp started with a pronounced compound IPSP of up to −5 mV amplitude. More insight into coupling of membrane hyperpolarization and subsequent excitation was provided by spontaneous synaptic activity, as well as hyperpolarizing current injection. After a singing episode, we recorded a continuous train of IPSPs (Fig. 9D). The individual IPSPs had amplitudes between −2 and −5 mV (average: −3.1 mV; *N* = 1, *n* = 30; asterisk in Fig. 9D inset), occurred at a rate of 15–20 Hz, and were followed by transient postinhibitory depolarization of 10–20 msec duration and peak amplitudes of 0.3–1.1 mV (average: 0.6 mV; *N* = 1, *n* = 30; arrowhead in Fig. 9D inset). Similarly, hyperpolarizing current injection of −2 nA for 500 msec elicited a subsequent rebound depolarization of 4 mV (peak amplitude), which triggered an immediate spike response and rhythmic singing activity starting about 300 msec after the stimulation (Fig. 9D). Short (125 msec) hyperpolarizing current pulses of −4 nA also entailed rebound depolarization that reliably triggered a single spike that was frequently followed by 1–3 IPSPs after 100–200 msec (Fig. 9E). When hyperpolarizing pulses (−4 nA; 125 msec) were injected repetitively at 2 Hz, corresponding to a slow chirp rate, they eventually triggered brief episodes of rhythmic membrane potential oscillation accompanied by singing motor activity. To quantify the relation between hyperpolarization and subsequent rebound depolarization, the closer interneuron was stimulated with hyperpolarizing current pulses of different amplitudes but with a constant duration of 125 msec. No depolarization or spike response occurred after stimulation with −1 nA (*N* = 1, *n* = 5), whereas pulses of −3 nA (*N* = 1, *n* = 5) evoked 1–3 mV poststimulus depolarizations that occasionally triggered a single action potential. Current pulses of −4 nA (*N* = 1, *n* = 5) elicited rebound depolarizations of 2–4 mV that reliably triggered 1–2 spikes (see average responses in Fig. 9F). The post-hyperpolarization spike response was frequently accompanied by consecutive IPSPs occurring after 100–300 msec (Fig. 9E).

In another cricket, a recording from the dendrite of a closer interneuron in the unfused abdominal ganglion A3 (data not shown) showed very similar characteristics. This closer neuron also received a volley of 4–6 IPSPs immediately before each singing episode and transient hyperpolarizing current injection (−0.5 nA) in the dendrite of the neuron triggered enduring singing activity with the normal chirp pattern. During the first chirps of a singing episode, its overall membrane potential slowly hyperpolarized and after singing stopped it repolarized within 3–5 sec to the resting potential. During singing, the neuron hyperpolarized by 5–10 mV in phase with the opener-motoneuron activity and depolarized by 10–15 mV in phase with the closer motoneurons. Each depolarization gave rise to a burst of 2–4 action potentials starting 9.3 ± 0.9 msec (mean ± SD; *N* = 1, *n* = 50) after the beginning of the wing-opener activity and 14.3 ± 0.9 msec (mean ± SD; *N* = 1, *n* = 50) before the wing-closer activity, which is 4–6 msec earlier than the closer neurons we recorded in the abdominal neuromeres of the metathoracic ganglion.

## Discussion

The neural basis of cricket singing has been repeatedly the subject of neurobiological studies (reviews: Kutsch and Huber 1989; Elsner 1994; Gerhardt and Huber 2002). Here, we intracellularly recorded and stained interneurons of the singing network and demonstrated their impact on singing pattern generation by intracellular current injection.

### Motor pattern of fictive singing

After cutting all wing nerves, fictive singing was evoked by microinjection of eserine in the brain neuropiles housing the dendrites of the descending calling song command neurons (Hedwig 2000). With a syllable cycle of 21–26 Hz and a chirp cycle of 2.3–2.9 Hz, the fictive singing motor pattern precisely matched the temporal characteristics of the natural calling song (Doherty 1985; Verburgt et al. 2011). Even minute details like the gradual decrease in the instantaneous syllable rate within the chirps and the constant temporal coupling between wing-opener and wing-closer activity (Kutsch 1969) remained unchanged after deafferentation. This clearly demonstrates that in contrast to locomotory pattern generators (Pearson 1995; Ausborn et al. 2007; Büschges and Gruhn 2008), the cricket singing CPG operates independent of sensory feedback to produce a characteristic and highly stable motor pattern, as required for species-specific signaling. Also in intact crickets, the circuitry of the singing network dictates the temporal pattern of the calling song, whereas mechanosensory feedback merely adjusts the precise angular position and closing velocity of the moving wings (Möss 1971; Elliott 1983; Schäffner and Koch 1987) to ensure a proper engaging force for sound production (Elliott and Koch 1983).

### Organization of the singing network

**Figure 10:**
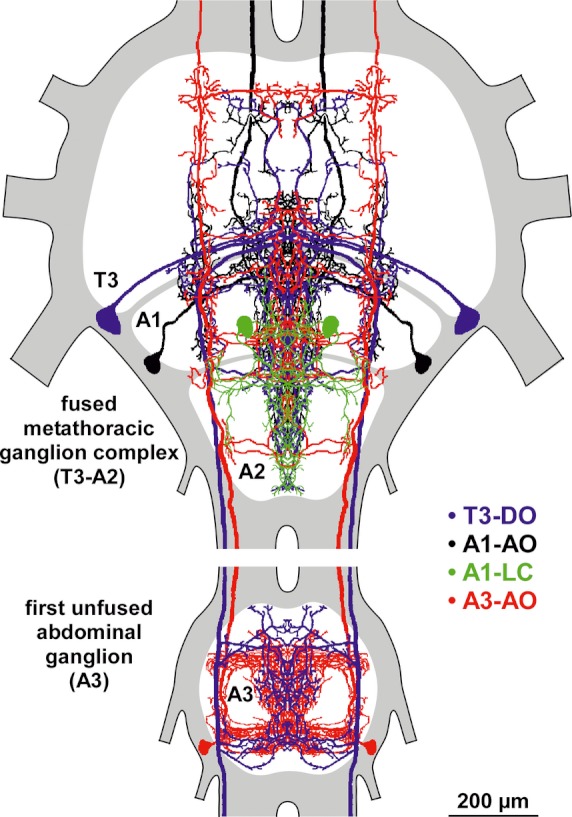
Overlay drawing of dendritic and axonal arborizations of singing interneurons in the metathoracic ganglion complex and abdominal ganglion A3. The conspicuous concentration of arborizations in the dorsal midline neuropiles of the metathoracic and first three abdominal neuromeres point toward these neuropiles as the location for singing pattern generation.

All singing interneurons we identified exhibited characteristic arborizations in the dorsal midline neuropiles of the fused metathoracic and first unfused abdominal ganglion (Fig. 10; Table 1). Likewise, previously identified singing interneurons had dendrites projecting posteriorly along the midline of the metathoracic ganglion complex (Hennig 1990). This local clustering of intermeshing singing interneuron arborizations clearly indicates the dorsal midline neuropiles of the metathoracic and three most anterior abdominal neuromeres as the location of singing pattern generation. Initially the CPG was thought to be located in the mesothoracic ganglion, which houses the singing motoneurons (Kutsch 1969; Kutsch and Otto 1972; Hoy 1978). Our data, however, now confirm at the cellular level the previously indicated spatial separation between the ganglion that generates the final motor output and the ganglia housing the CPG (Hennig and Otto 1995; Schöneich and Hedwig 2011) by revealing crucial CPG interneurons in A3, which had not been described in detail before.

**Table 1:** Singing interneurons in *Gryllus bimaculatus*

Interneuron	Number of specimen recorded/stained	Action potentials per syllable	Interneuron spike delay… (mean ± SD in msec)	Reset of the chirp rhythm
Opener INs			…to wing-opener motoneurons	
T3-DO	17/10	3–4	7.0 ± 0.8	Yes
A1-AO	1/1	3–6	7.5 ± 1.1	
A3-AO^1^	12/8	5–6	10.1 ± 0.8	Yes
Closer INs			…to wing-closer motoneurons	
A1-LC	1/1	2–4	10.2 ± 1.1	
cIN in A2	1/–	6–7	8.0 ± 0.4	Yes
cIN in A3	1/–	2–4	14.3 ± 0.9	

1 Some preliminary data of this neuron had been reported in a short communication (A3-IN; Schöneich and Hedwig 2011).

In grasshoppers, which use their hind legs for sound production, singing interneurons with reset properties also have characteristic medial arborizations in the dorsal neuropile of the metathoracic–abdominal ganglion complex (Gramoll and Elsner 1987; Hedwig 1992; Schütze and Elsner 2001). Despite the use of different thoracic appendages (hind legs vs. front wings), in grasshoppers as well as in crickets, the singing network extends over the same neuromeres (T3 and A1–A3). Also in Drosophila, typical wing vibrations of male courtship singing can be elicited by stimulation of specific thoracic–abdominal interneurons (Clyne and Miesenböck 2008; von Philipsborn et al. 2011) and in arctiid moths that use tymbals for rhythmic sound production, the motor pattern is generated in the thoracic–abdominal ganglion complex as well (Dawson and Fullard 1995). This suggests that the circuits for intraspecific acoustic signaling have a common evolutionary origin based on early thoracic–abdominal motor control networks, which may have been linked to ventilation (cf. Robertson et al. 1982; Dumont and Robertson 1986).

Interestingly, the morphology of T3-DO in the metathoracic ganglion as well as its descending axon with projections in every unfused abdominal ganglion resembles the ventilation-coordinating interneurons identified in locusts (Pearson 1980; Ramirez and Pearson 1989). Considering that in a singing cricket, the abdominal ventilation cycles are strictly coupled to the chirp rhythm (Paripovic et al. 1996), the axonal projections of T3-DO in the posterior abdominal ganglia could link the singing CPG output to the abdominal ventilatory oscillators (Kammer 1976; Ramirez and Pearson 1989).

### Cellular organization of the singing CPG

In accordance with other studies (Robertson and Pearson 1983; Ramirez and Pearson 1989; Jürgens and Hage 2007), we only considered an interneuron to be part of the singing CPG if its rhythmic spike discharge strictly preceded the motor activity, and if transient perturbation of its activity reset the fictive singing pattern. In terms of these criteria, the opener-interneuron A3-AO and T3-DO and the closer interneurons recorded in A2 and A3 qualify as components of the singing CPG.

The ascending and descending opener-interneuron A3-AO and T3-DO occurred both as pairs of bilateral mirror-image sibling cells, and in both cases, intracellular depolarizing current injection in either the right or left interneuron was sufficient to elicit singing motor activity. If the singing CPG consists of bilateral-symmetrical hemioscillators (Ronacher 1989; Hennig and Otto 1995), at some point the left and right subcircuits need to be coupled to ensure coordinated movement of the two forewings for sound production. A common mechanism for synchronizing CPG neurons is electrical coupling via gap junctions (Marder and Calabrese 1996; Kiehn and Tresch 2002), which is often indicated by dye coupling (Ewadinger et al. 1994; Antonsen and Edwards 2003; Fan et al. 2005). Labeling an A3-AO with neurobiotin reliably stained the contralateral A3-AO sibling neuron as well, whereas for T3-DO even intense neurobiotin labeling never indicated any dye coupling. This points toward electrical synapses between the A3-AO sibling cells providing bilateral synchronization of the motor pattern. Besides graded synaptic transmission (Simmons 1982; Manor et al. 1997), electrical coupling would explain how subthreshold shifting of the A3-AO membrane potential modulated the singing rhythm (cf. Mulloney et al. 1981; Mamiya et al. 2003). Similar subthreshold interaction has been reported between flight CPG neurons in the locust (Robertson and Reye 1988).

The spatial overlap of the T3-DO main dendrite with axonal arborization of both A3-AO neurons and vice versa (Fig. 10) indicate mutual synaptic connections between these CPG neurons. As spike activity in the ascending A3-AO neurons strictly preceded the first T3-DO spike by about 3 msec, the depolarization of T3-DO could be driven by excitatory A3-AO inputs, whereas the depolarization of A3-AO cannot primarily result from descending T3-DO inputs and may involve the descending command neurons.

Some flight CPG interneurons directly activate motoneurons (Robertson and Pearson 1985). As mesothoracic and prothoracic motoneurons contribute to singing (Kutsch 1969; Pfau and Koch 1994), the meso- and prothoracic axon collaterals of A3-AO may allow such direct connections. The opener-inter neuron A1-AO forward the rhythmic CPG output from the metathoracic ganglion to the mesothoracic motor network without interfering with pattern generation.

### Generation of syllable and chirp rhythm

Our experiments clearly indicate A3-AO and T3-DO as crucial elements of the syllable–rhythm-generating network (cf. Figs. 2C–E and 6C–E). The membrane potential oscillations in A3-AO and T3-DO seem to result from excitatory inputs as well as inhibitory connections with yet unidentified closer interneurons like those we recorded in the anterior abdominal neuromeres. Besides the ability to reset the singing motor pattern, the closer interneuron we recorded in A2 (Fig. 9) received substantial inhibition in the opener phase and also exhibited postinhibitory rebound depolarization. Mutual inhibiting interneurons that respond with postinhibitory rebound generate rhythmically alternating activity bursts (Perkel and Mulloney 1974; Satterlie 1985). Our data indicate that rebound from opener-phase inhibition triggers closer-interneuron spiking, which in turn temporarily inhibit opener interneurons during the closer phase. This would explain the tight latency coupling of wing-opener and wing-closer bursts in the motor pattern (Kutsch and Huber 1989). Only opener interneurons (e.g., A3-AO) showed small subthreshold depolarizations following the last syllable cycle of the chirps, whereas in closer interneurons the depolarization of the last syllable in the chirp slowly decayed (e.g., Fig. 9A). This suggests that once a chirp has started, the alternate bursting of opener and closer interneurons continues until the opener neurons finally fail to generate a spike burst.

Activated by tonic command neuron spike activity, the singing CPG generates the species-specific calling song pattern with 3–5 syllables grouped to chirps. Constant depolarizing current injection in A3-AO or T3-DO, however, elicited sustained syllable trains, which reliably reset the ongoing chirp rhythm. Subsequent to current elicited syllables, the next chirp always started after a regular chirp interval. This result contradicts the idea of an independent chirp-cycle generator that periodically drives or inhibits the syllable generating circuit (Kutsch 1969; Bentley 1969). The chirp rhythm rather originates from activity-dependent inherent network and/or cellular properties (Bentley and Hoy 1972) that regularly silence the syllable generation and let it recover after a normal chirp interval. Additionally, the chirp pattern is stabilized by rhythmic feedback loops comprising interneurons of the subesophageal and posterior abdominal ganglia (Otto and Hennig 1993; Schöneich and Hedwig 2011) and also depends on the activity level of the descending command neurons (Hedwig 2000). Further studies are required to reveal the neural mechanism controlling chirp generation, which probably include activity-dependent slow changes of membrane conductances (El Manira et al. 1994; Harris-Warrick 2010) and/or periodical recovery of strongly depressing synapses (Manor and Nadim 2001).

### Future prospects

Our identification of singing CPG neurons in *Gryllus bimaculatus* highlights the importance of ganglion A3. The data provide the basis for further studies to establish the functional circuitry of the network and to reveal evolutionary modifications in the singing CPG that account for the distinctive calling song patterns in related cricket species (Alexander 1962). Moreover, the connection between the singing CPG and the corollary discharge interneuron that modulates auditory processing during sound production (Poulet and Hedwig 2002, 2006) may now be targeted.

As gene expression studies considerably benefit from better knowledge about where in the nervous system the relevant phenotypic differentiations are most likely to occur (Shaw and Danley 2003), our results will also contribute to research efforts to obtain a genic understanding of speciation in crickets (Ellison et al. 2011) and also other acoustically communicating insects such as Drosophila (Rideout et al. 2007; von Philipsborn et al. 2011).
